# Growth Differentiation Factor 15 as a Link Between Obesity, Subclinical Atherosclerosis, and Heart Failure: A Systematic Review

**DOI:** 10.3390/medicina62010132

**Published:** 2026-01-08

**Authors:** Raluca-Elena Alexa, Alexandr Ceasovschih, Bianca Codrina Morărașu, Andreea Asaftei, Mihai Constantin, Alexandra-Diana Diaconu, Anastasia Balta, Raluca Ecaterina Haliga, Victorița Șorodoc, Laurențiu Șorodoc

**Affiliations:** 1 Grigore T. Popa University of Medicine and Pharmacy Iasi, 700115 Iasi, Romania; 2 2nd Internal Medicine Clinic, ‘Sf. Spiridon’ Clinical Emergency Hospital, 700111 Iasi, Romania

**Keywords:** obesity, growth differentiation factor 15, GDF-15, atherosclerosis, heart failure

## Abstract

*Background and Objectives*: Obesity, heart failure (HF), and atherosclerosis have common pathways, including chronic inflammation, immune cells activation, and metabolic disturbances. These pathways often coexist and overlap, increasing cardiometabolic risk. Growth differentiation factor 15 (GDF-15) is an emerging cytokine linked to inflammation, oxidative stress, and metabolic dysregulation, which are common pathways between heart failure, obesity and atherosclerosis. Beyond its established prognostic value in cardiovascular diseases (CVD) and HF, recent evidence suggests that GDF-15 may also reflect subclinical atherosclerosis, potentially improving early risk stratification in obese and HF populations. The aim of this review is to synthesize current evidence on the association between GDF-15 and markers of subclinical atherosclerosis, and to evaluate whether GDF-15 may serve as an integrative biomarker reflecting shared cardiometabolic pathways. *Materials and Methods*: We conducted a systematic review following PRISMA recommendations registered by CRD420251267457 number on PROSPERO. PubMed, Embase, Scopus, and Web of Science were searched for human studies evaluating the correlation between markers of subclinical atherosclerosis and GDF-15 concentration. We excluded the studies not published in English, not involving human participants, and not meeting the inclusion criteria. We assessed the risk of bias using the Joanna Briggs Institute appraisal tool. Due to the heterogeneity of studies, a narrative synthesis was performed. *Result*: The review included 18 studies, which evaluated the association between GDF-15 and subclinical atherosclerosis markers, such as intima media thickness, coronary artery calcium score, ankle-brachial index, and atherosclerotic plaques. Studies included patients with metabolic disorders, chronic inflammatory diseases, HIV cohorts, and general population samples. Most of the studies reported that GDF-15 levels were associated with greater atherosclerotic burden; however, results were frequently influenced by confounders. Methodological limitations, such as limited or highly specified samples, cross-sectional designs, variability in atherosclerotic-imaging technique, and inconsistent adjustment for confounders, restrict generalization of the results. *Conclusions*: Current evidence supports GDF-15 as a biomarker integrating inflammatory and metabolic stress signals, indirectly linking obesity, HF and subclinical atherosclerosis. While current data supports its prognostic relevance, further studies are needed to confirm its clinical utility in routine assessment and preventive cardiovascular care.

## 1. Introduction

Obesity, subclinical atherosclerosis, and heart failure (HF) share common pathways influenced by chronic inflammation, oxidative stress, endothelial dysfunction, and metabolic disturbances. Increased adiposity promotes insulin resistance, activates immune and inflammatory mediators, and induces atherogenic dyslipidemia, ultimately contributing to metabolic syndrome and end-organ dysfunction [1,2,3].

Worldwide, cardiovascular diseases (CVD) remain the leading cause of mortality and morbidity, with obesity rising dramatically among individuals in their third to fifth decade of life. This trend contributes to the increasing burden of cardiometabolic disorders and supports the need for early prevention and the identification of subclinical disease states [4,5].

In recent years, GDF-15 has emerged as a promising biomarker, as it is involved in many of the pathological mechanisms underlying obesity, HF, and atherosclerosis [6,7]. GDF-15 is a stress-responsive cytokine, triggered by inflammation, mitochondrial dysfunction, hypoxia, insulin resistance, and endothelial injury [8,9]. Moreover, elevated GDF-15 levels have a strong prognostic value for cardiometabolic disorders and all causes mortality, in addition to traditional biomarkers [8,10,11,12].

Although several studies have explored the relationship between GDF-15 and obesity, atherosclerosis, or heart failure, no study has simultaneously assessed all three conditions together. This is a knowledge gap, as these disorders frequently coexist and share common pathways.

The aim of this review is to synthesize recent evidence on the relationship between GDF-15 and different subclinical atherosclerosis markers, and to evaluate whether GDF-15 may represent an integrative biomarker reflecting shared pathways between atherosclerosis, obesity, and heart failure.

## 2. Methods

We conducted a systematic review of the literature regarding the association between GDF-15 and subclinical atherosclerosis. We registered the review protocol in PROSPERO under the registration number CRD420251267457, and developed our study strategy based on the Population, Intervention, Comparison, Outcome, and Study design strategy, using the PRISMA statement for systematic reviews [13].

### 2.1. Research Question and Search Strategy

We conducted systematic literature research in PubMed, Scopus, Embase, and Web of Science, from database inception to 30 November 2025. Search strategies included MeSH and Emtree controlled vocabulary terms, free text terms, such as “growth differentiation factor-15”, ankle-brachial index, intima media thickness, coronary artery calcium score, and subclinical atherosclerosis. We used search syntax such as (“GDF-15” OR “growth differentiation factor-15”) AND (“ankle brachial index” OR “ ABI”), (“GDF-15” OR “growth differentiation factor-15”) AND (“coronary artery calcium score” OR “CACS”), (“GDF-15” OR “growth differentiation factor-15”) AND (“intima-media thickness” OR “ IMT”), (“GDF-15” OR “growth differentiation factor-15”) AND (“subclinical atherosclerosis”, and obtained 57 articles from PubMed, 51 articles from Scopus, 96 articles from Embase, and 198 articles from Web of Science.

### 2.2. Inclusion Criteria

The inclusion criteria were original full-text articles published until 30 of November 2025, conducted on human populations, including randomized controlled trials, clinical trials, and cross-sectional, observational, and cohort studies that studied the association between GDF-15 serum levels and subclinical atherosclerosis.

### 2.3. Exclusion Criteria

The exclusion criteria included case report articles, reviews, systematic reviews, meta-analyses, letters to the editors, articles published in languages other than English, or articles from pre-clinical studies. We also excluded studies conducted in patients younger than 18 years old.

### 2.4. Study Selection

Studies that met eligibility criteria (1) included human participants; (2) evaluated the correlation between GDF-15 serum levels and subclinical atherosclerosis marker; (3) provided statistical results such as *p*-values. Studies were excluded if they (1) were other types of articles (such as reviews, meta-analyses, abstracts, posters, etc.); (2) provided insufficient data; (3) did not correlate GDF-15 with a subclinical atherosclerosis marker; (4) included patients with clinical atherosclerosis; (5) did not assess GDF-15 serum levels.

**Figure 1:**
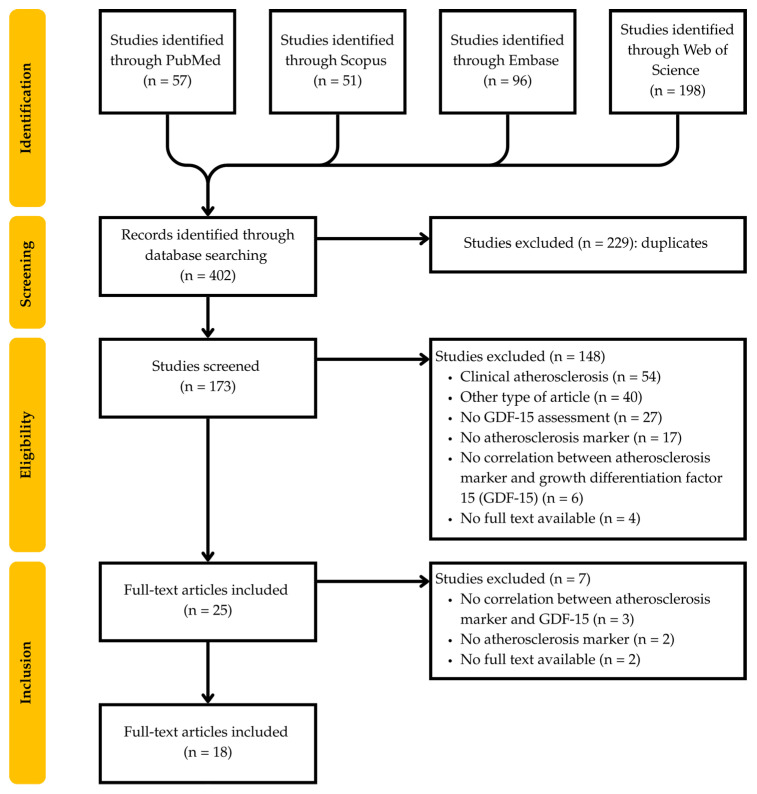
PRISMA flow chart.

The study selection process is detailed in Figure 1. From 402 studies initially identified, 229 were duplicates, and 155 were excluded based on exclusion criteria. We identified 18 eligible studies, including 14 cross-sectional studies, 3 cohort studies, and 1 case–control study, covering metabolic disorders, chronic inflammatory diseases, HIV cohorts, elderly or general population, and other disorders such as chronic kidney disease or beta-thalassemia.

### 2.5. Data Extraction

Two independent researchers screened the articles to identify correlations between at least one subclinical atherosclerosis marker and GDF-15 serum levels, and, additionally, for correlations between GDF-15 and body mass index, obesity, metabolic profile, heart failure, and cardiovascular risk. Other relevant information regarding the association between obesity, heart failure, and subclinical atherosclerosis was noted. This process was standardized by including information about the study design, study population, aim, inclusion and exclusion criteria, and outcomes. If any disagreements occurred, they were settled by a third reviewer.

### 2.6. Risk of Bias Assessment

Risk of bias was assessed using the Joanna Briggs Institute (JBI) appraisal tool [14], namely the JBI Checklist for analytical cross-sectional studies, JBI Checklist for case–control studies, and JBI Checklist for cohort studies. All the items were judged as Yes/No/Unclear/Not applicable, according to the instructions. The purpose of this assessment was to evaluate the methodological quality, the validity of biomarker and imaging measurements, identification and control of confounding, and statistical adequacy.

Table 1, Table 2 and Table 3 summarize the risk of bias assessment for included studies. Most of the studies clearly defined the eligibility criteria, described the study population, used validated methods for GDF-15 serum levels measurements, and standardized protocols for subclinical atherosclerosis assessment. Regarding possible bias, some studies had partial or no adjustment for confounders, leading to some uncertainty. Furthermore, no major concerns were identified regarding exposure or outcome, meaning that the principal source of bias relates to residual confounding rather than measurement error.

### 2.7. Strategy of Data Synthesis

After the selection process, 18 studies were included in this systematic review. A narrative synthesis of the findings was performed, focusing on the association between GDF-15 serum levels and subclinical atherosclerosis in human population.

Table 4 summarizes the correlation between GDF-15, subclinical atherosclerosis, and, where available, correlations between GDF-15 and heart failure or cardiovascular risk, metabolic profile (obesity or lipid profile), and other findings relevant to our aim.

To our knowledge, no study has directly evaluated the association between GDF-15 and subclinical atherosclerosis in patients with obesity and heart failure. Therefore, the results regarding this population are indirect and derive from studies in which GDF-15 also correlates with body mass index or heart failure. These findings may support the hypothesis of a potential role of GDF-15 involvement in subclinical atherosclerosis in patients with obesity and heart failure. However, this extrapolation should be interpreted with caution due to the lack of dedicated studies.

## 3. Results

Table 4 summarizes the clinical studies regarding GDF-15 association with subclinical atherosclerosis in patients with metabolic inflammatory, infectious, and renal disorders, along with elderly and general population. The subclinical atherosclerosis markers were ankle-brachial index, coronary artery calcium score, carotid intima-media thickness, and atherosclerotic plaque measurement.

### 3.1. Results from Studies

#### 3.1.1. Metabolic Disorders

Across metabolic conditions, GDF-15 was associated with multiple markers of subclinical atherosclerosis, such as ABI, CIMT, or FIMT. Typically, these populations exhibit visceral adiposity which induces insulin resistance and chronic low-grade inflammation, these mechanisms triggering GDF-15 expression. Moreover, the relationship between GDF-15 and different metabolic parameters such as HOMA-IR or steatosis score, connects GDF-15 to metabolic disturbances beyond adiposity [17,20]. Since within these cohorts, GDF-15 appears to capture metabolic and vascular stress, it could be a promising biomarker for early vascular disease diagnosis in patients with risk for cardiometabolic complications.

#### 3.1.2. Chronic Inflammatory Diseases

In inflammatory diseases, the main cause of atherosclerotic disease is endothelial dysfunction, which is the result of different mechanisms. In patients with chronic obstructive pulmonary diseases, elevated GDF-15 levels may be explained by intrathoracic mechanics and hypoxia [24]. In contrast, chronic inflammation primarily triggers for GDF-15 expression in psoriasis [21], and immunosuppression contributes in rheumatoid arthritis [26], leading to endothelial dysfunction and pro-atherogenic processes. Furthermore, in antiphospholipid syndrome, the main mechanism is represented by impaired nitric oxide release, which eventually leads to endothelial dysfunction [27]. Overall, GDF-15 reflects vascular injury induced through different pathways.

#### 3.1.3. Human Immunodeficiency Virus (HIV) Cohorts

In patients with HIV, the studied cohorts were heterogenous. In younger patients, with low cardiovascular risk, GDF-15 was not associated with atherosclerosis [15,33], whereas in older, metabolically compromised, where GDF-15 was associated with plaque volume [25]. The differences between groups could be explained by age, antiretroviral therapy duration, metabolic health, and systemic inflammation, which are more frequent in older patients and modulate the GDF-15-atherosclerosis association in HIV populations. Thus, GDF-15 appears useful primarily in high-risk HIV patients.

#### 3.1.4. General Population

In the general population and elderly patients, GDF-15 was frequently associated with atherosclerotic plaque [16,18,19,23] and a coronary artery calcium score [22,30]. Moreover, genetic variants were associated with atherosclerotic plaque and risk of T2DM [19], and age was correlated with N-terminal pro-B-type natriuretic peptide [16,23,30]. These results suggest that mitochondrial dysfunction, oxidative stress, and systemic inflammation induced by aging are responsible for GDF-15 increased levels, being a good predictor for mortality and atherosclerotic events in the elderly.

#### 3.1.5. Other Diseases

In patients with chronic kidney disease (CKD), chronic inflammation status and uremic toxicity lead to vascular injury, being associated with CIMT. Furthermore, GDF-15 reflects a combination between metabolic, vascular, and uremic stress, being correlated with cardiovascular risk [31].

In patients with beta-thalassemia, the association between CIMT and GDF-15 levels was significant. Although pathogenic mechanisms differ from typical cardiometabolic disease, oxidative stress leads to endothelial dysfunction and vascular inflammation, stimulating GDF-15 expression.

### 3.2. Effect Direction and Magnitude

In most of the studies, the association between GDF-15 and markers of subclinical atherosclerosis was positive, and directly correlated with atherosclerotic burden [16,17,18,19,20,21,22,23,24,25,26,27,30,31,32]. This pattern was observed in patients with metabolic disorders, chronic inflammatory diseases, the general population, elderly individuals, and patients with chronic kidney disease, supporting the relationship between systemic inflammation and endothelial dysfunction. In contrast, in patients with HIV and younger individuals, the association was weak or absent, indicating the fact that the effect depends on the underlying condition. In studies where adjustment for confounders was possible, the positive association was mostly preserved, but decreased, further suggesting that confounders contribute to, but do not explain, the results.

### 3.3. Heterogeneity

The correlation between GDF-15 concentrations and markers of subclinical atherosclerosis showed a significant variability among different cohorts. The association was strongest in elderly patients, in those with chronic inflammatory disorders, and those with chronic kidney disease, where GDF-15 correlated with CIMT, atherosclerotic plaque, or coronary calcification. For these situations, the association remained statistically significant after adjustment for traditional risk factors, indicating the cumulative influence of inflammation, oxidative stress, and metabolic disturbances [18,19,22,23,24,25,27,30,32].

In metabolic disorders, GDF-15’s association with subclinical atherosclerosis was present, but inconsistent, since this association tended to be stronger in patients with advanced metabolic disturbances, and weaker in younger patients or those with better metabolic control [17,20,34].

In contrast, in HIV cohorts, which were generally younger patients with lower cardiovascular risk, the association was weaker [25] or absent [15,33]. After adjustment for confounders, the previously positive association was attenuated or lost, suggesting that in lower-risk settings, GDF-15 may reflect background systemic stress rather than atherosclerotic process directly.

Taken together, these findings support the idea that the association between GDF-15 and subclinical atherosclerosis is context-dependent, being most evident in situations involving inflammation, metabolic imbalance, vascular aging, or impaired renal function, all of these being risk factors for atherosclerosis.

Interpretation of statistical analysis must consider methodological heterogeneity. First, GDF-15 was measured using ELISA in most studies, but also other types of immunoassay platforms [15,16,18,21,22,27] or PCR [19]. Second, the results were reported different units, and data were analyzed using absolute value, log-transformed scales, or tertiles. Third, the atherosclerosis assessment varied, and the imaging protocols were different. All these factors limit direct comparability of effect data, despite consistent directional trends.

### 3.4. Evidence Gaps

Despite GDF-15 relevance in cardiometabolic disorders, several gaps still remain regarding its implication in the triad of obesity–heart failure–subclinical atherosclerosis. First, no study simultaneously evaluated its implication in the triad mentioned above, which is the main interest for this review. Therefore, the results are indirect and derived from different and heterogeneous populations, and the conclusions are not easy to generalize.

Second, the majority of studies are cross-sectional, which limit causality. Moreover, the incremental predictive value of GDF-15 beyond established cardiovascular risk factors has been evaluated in only few cohorts, and the results are not conclusive.

Third, the heterogeneity of studies, reflected in differences in vascular imaging protocols, statistical analysis, or endpoints, makes it more difficult to compare study results, and contribute to discordant findings. Confounders such as age, renal impairment, chronic inflammation, metabolic dysfunction, and cardiovascular risk remain key determinants in GDF-15’s involvement in both cardio–reno–metabolic disorders and atherosclerosis.

Finally, few studies differentiated between heart failure phenotypes when evaluating GDF-15’s involvement, particularly in patients with obesity, where it may have greater diagnostic and prognostic significance.

Overall, there is a need for dedicated studies, with standardized protocols for vascular assessment, clear endpoints, and statistical analysis to clarify whether GDF-15 represents a marker of systemic stress or an integrative biomarker, with independent clinical utility in cardiometabolic disorders.

## 4. Discussion

### 4.1. GDF-15 Physiopathological Implications

**Figure 2:**
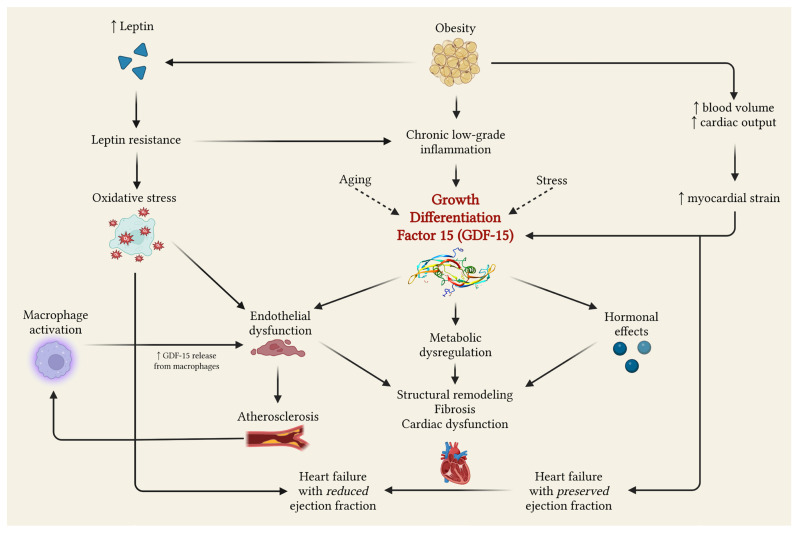
GDF-15 shared pathways with obesity, heart failure, and atherosclerosis. Created with BioRender. Ceasovschih, A. (2025). https://BioRender.com/e2ri1wf (accessed on 26 December 2025).

GDF-15 is a member of the transforming growth factor-β (TGF-β) superfamily and is involved in several biological processes, including oxidative stress response, weight management, inflammation, and cancer progression [7,35]. Circulating levels of GDF-15 increase with age, suggesting its role as a systemic biomarker of age-related diseases, including cardiometabolic conditions [8,10,36,37] (Figure 2).

#### 4.1.1. Tissue Expression and Stress Responsiveness

GDF-15 is expressed in various tissues, including the heart, lung, colon, liver, kidney, vascular smooth muscle cells, and endothelial cells. Under stress condition, its expression increases, highlighting its role as a stress-response cytokine and its utility as a diagnostic, prognostic, and potentially therapeutic biomarker [8,38,39].

#### 4.1.2. Molecular Regulation

At the molecular level, GDF-15 transcription is regulated through pathways, such as p53, EGR-1, strongly linking it to cellular damage, senescence, and immunomodulation [11,36].

Serum levels are typically low in young and healthy individuals, but they rise progressively with age, modulating the subclinical development of cardiometabolic disorders. Moreover, GDF-15 exerts autocrine, endocrine, and paracrine effects depending on the tissue involved and the stage of disease [10,36,37].

#### 4.1.3. GFRAL-RET Axis and Metabolic Effects

According to the studies, GDF-15’s effect is mediated through a complex formed by the glial cell-derived neurotrophic factor family receptor alpha-like (GFRAL) and a tyrosine kinase receptor (RET), located in area postrema and nucleus tractus solitarius. Through this pathway, GDF-15 exerts an anorexigenic effect, reducing appetite and food intake, which confers a central role in influencing body weight and energy expenditure [40,41]. Murine studies have demonstrated that GFRAL-RET agonism improves metabolic parameters, while its antagonism leads to weight gain and metabolic dysfunctions [8,42].

### 4.2. Diagnostic and Prognostic Role of GDF-15

Although elevated GDF-15 levels are not specific to a single condition, the molecule is considered a valuable biomarker of global disease burden and systemic stress rather than a disease-specific indicator [8,10]. Its strongest evidence base lies in CVD, where increased levels are found in hypertension, atherosclerosis, heart failure, especially with preserved ejection fraction, and peripheral artery disease. In these conditions, GDF-15 serves as a prognostic biomarker, and is independently associated with cardiovascular events and mortality beyond traditional risk markers [9,36,43,44]. However, its diagnostic value is limited due to confounders, such as age, renal impairment [30,31], metabolic disturbances, and inflammation [21,27], which decrease its specificity for individual conditions.

Because its expression is stress-related and amplified by inflammation, elevated levels are also encountered in patients with obesity, insulin resistance, type 2 diabetes mellitus (T2DM), or steatotic liver disease [9,34,36]. Moreover, GDF-15 levels rise with aging, and are strongly correlated with frailty and physical function decline in older adults [11,37,45]. Accordingly, recent research describes GDF-15 as a molecule situated at the crossroads of inflammation, metabolism, and aging. Its utility in risk stratification and phenotyping is particularly enhanced when integrated into multimarker strategies along with biomarkers such as NT-proBNP or hs-CRP, improving patient classification, and therefore supporting its prognostic value rather than diagnostic. [8,10,11,12,30],

### 4.3. Possible Mechanisms Between Atherosclerosis, Heart Failure and Obesity

Obesity induces chronic low-grade inflammation, which increases the secretion of proinflammatory cytokines such as tumor necrosis factor α (TNF-α), and chemokines, such as GDF-15. These molecules induce endothelial dysfunction and insulin resistance, perpetuating the inflammatory state and driving immunological dysfunction through activation of immune system cells [2,46,47,48].

Hemodynamically, obesity increases blood volume and cardiac output, contributing to left ventricular hypertrophy and diastolic dysfunction, features of HF with preserved ejection fraction (HFpEF). I would like to rephrase it: Furthermore, obesity leads to structural heart changes and HF with reduced ejection fraction (HFrEF) by activating neurohormonal pathways, especially through leptin [46,49]. Paradoxically, in spite of the elevated leptin levels in obese patients, leptin resistance further contributes to molecular mechanisms in oxidative stress, endothelial dysfunction, and chronic inflammation. All these promote and sustain the atherosclerotic process [50,51].

In atherosclerosis, macrophage activation facilitates the uptake of oxidized low-density lipoprotein and secretion of GDF-15, contributing to the atherosclerotic plaque formation and to the local inflammation [8,46,52]. As a consequence, GDF-15 is released from macrophages within atherosclerotic plaques and correlates with vascular inflammation, promoting plaque progression and stenosis [1,48]. Despite inconsistencies in published findings, most studies suggest an association between elevated GDF-15 and subclinical or progressive atherosclerosis, as well as with cardiovascular outcomes [8].

Chronic HF represents the final common pathway of hemodynamic overload, metabolic disturbances, and inflammation, often originating from obesity and atherosclerosis. All these modifications promote structural remodeling, fibrosis, and progressive cardiac dysfunction [53,54]. According to contemporary studies, GDF-15 is a strong predictor of HF severity and mortality, reflecting hypoxia, oxidative stress, systemic inflammation, and mechanical strain [1,18]. Beyond its prognostic role, GDF-15 exerts immunomodulatory effects and may attenuate the maladaptive hypertrophy response [16,18].

In summary, GDF-15 integrates the major sources of cardiometabolic stress. It highlights metabolic and inflammatory response associated with obesity [2,46], vascular inflammation of atherosclerotic plaques [1,3], and mechanical, oxidative, and hypoxic stress driven to chronic HF progression [18,53]. Thus, GDF-15 can be considered a biomarker capable of quantifying the interaction between obesity, atherosclerosis, and HF.

### 4.4. GDF-15 as Pathophysiological Mediator vs. Risk Biomarker

Since there are several mechanisms that could link obesity, heart failure, and atherosclerosis through GDF-15, an important question remains: should GDF-15 be interpreted as a risk biomarker or an active mediator in this triad?

All the mechanisms explained before position GDF-15 as a molecule capable of influencing vascular remodeling, plaque development, and myocardial structure and function. However, clinical evidence supports GDF-15 more strongly as a risk marker than a mediator. In most studies included in this review, the association between GDF-15 and subclinical atherosclerosis decreases after adjustment for confounders. Moreover, it is difficult to establish whether GDF-15 elevation precedes vascular dysfunction or represents a primary driver leading to it.

To move from association to causality, evidence of temporality, dose–response relationships, consistency across populations and methods, and ideally genetic phenotyping is required, since modulation of GDF-15 signaling could be influenced by the genetic profile.

Based on the current data available, GDF-15 appears to more likely be an integrative stress biomarker, capturing the influence of inflammation, metabolic dysregulation, endothelial dysfunction, and renal dysfunction. Whether it can act as a mediation in the triad of obesity–heart failure–atherosclerosis remains an open question that requires further studies.

### 4.5. Confounders

All results about the relationship between GDF-15 and subclinical atherosclerosis should be interpreted taking into account several important confounders. As we mentioned before, these include age, renal impairment, endothelial dysfunction, inflammation, and cumulative comorbidity burden. Table 5 summarizes the main confounders and the statistical results after adjustment (Table 5).

Age is one of the strongest determinants of GDF-15 serum levels, and correlates with endothelial dysfunction, and chronic comorbidities commonly associated with aging. Reduced renal function also increases GDF-15 serum concentration, particularly in patients with chronic kidney disease. In this population, uremia, inflammation, and malnutrition may explain the association between atherosclerosis and increased levels of GDF-15 [31].

Metabolic status plays an important role. Obesity, insulin resistance, diabetes, and fatty liver disease are positively associated with GDF-15 serum levels, reflecting metabolic and vascular stress, and endothelial dysfunction [18,19,21,24]. Additionally, frailty and chronic systemic disease also increase GDF-15 concentrations, independently of other confounders. Finally, in patients with HIV, antiretroviral treatment, along with anti-inflammatory agents or cardiometabolic medications, such as SGLT2 inhibitors, influence GDF-15 expression, but the results are inconsistent across studies [15,25,33].

After adjustment for confounders, the strength of the association between GDF-15 and atherosclerosis diminishes, and in some cases the statistical significance is lost [21,25,27]. Consistent, comprehensive confounder adjustment is mandatory in future studies, to ensure more accurate results and clinical interpretation.

### 4.6. Clinical Implications

Current evidence suggests that GDF-15 may serve as a global risk stratification and prognosis marker rather than a specific diagnostic marker. Elevated GDF-15 levels correlate with cardiometabolic risk, inflammation, and endothelial dysfunction and are linked to vascular disease. However, the inconsistent results limit at this moment its use in clinical practice, even after adjusting for confounders. GDF-15 is likely more effective when used alongside other biomarkers rather than alone. Moreover, the lack of longitudinal studies hinders the assessment of GDF-15’s diagnostic role for specific disorders. Further studies should focus on its role in specific populations, and assess its role in predicting the risk and treatment strategy.

### 4.7. Limitations

Across all included studies, the most common limitations were the predominance of cross-sectional designs, the use of small or highly specific samples, insufficient adjustment for confounders, and incomplete cardiovascular or metabolic evaluation, all of which may interfere with the results (Table 6). Several studies relied on self-reported medical histories, introducing measurement bias. Additionally, variation in imaging techniques contributes to heterogeneity, while differences in body composition across populations limited generalizability.

The heterogeneity across studies results from the differences in population characteristics, age, cardiovascular risk, and comorbidity burden. Methodological heterogeneity arose from differences in study design, imaging protocols applied to evaluate subclinical atherosclerosis, and statistical adjustment for confounders, especially renal function, systemic inflammation, malignancy, and metabolic factors.

In chronic inflammatory diseases, disease activity itself may influence circulating GDF-15, limiting the capacity to estimate the exact contribution of inflammation to GDF-15-atherosclerosis relationship.

In HIV cohorts, key variables such as viremia, antiretroviral therapy, and metabolic syndrome status were inconsistently reported. Many participants were relatively young and without CVD, reducing the probability of detecting GDF-15-atherosclerosis association.

Studies in beta-thalassemia and CKD on hemodialysis included small, highly specific samples, reducing external validity. In CKD, malnutrition status may elevate GDF-15, complicating interpretation.

In general population cohorts, most sources of bias included limited age ranges and voluntary enrollment, reducing sample representativeness. Missing information regarding BMI and HF association with GDF-15 limited the adjustment for confounders.

Taken together, these limitations restrict the capacity to determine causality and prevent a complete assessment of the role of GDF-15 across the triad of obesity, subclinical atherosclerosis, and HF.

### 4.8. Future Perspectives

To our knowledge, there is no study in the literature that has assessed GDF-15 involvement in subclinical atherosclerosis among patients with obesity and heart failure. Future research should aim to clarify whether GDF-15 serves as a biomarker that quantifies cardiometabolic stress across obesity and HF, and whether it can enhance early detection of subclinical atherosclerosis. Furthermore, investigations should integrate standardized vascular imaging, clinical evaluation of symptoms and comprehensive metabolic, inflammatory, and cardiac phenotyping.

The relationship between GDF-15 and HF, especially HFpEF in the context of obesity, requires focused investigation, as metabolic inflammation and myocardial strain may independently influence GDF-15 expression. In addition, molecular studies should examine how GDF-15 links mitochondrial dysfunction, endothelial damage, and neurohormonal activation. Given the increasing significance of the GFRAL-RET pathway, interventional studies targeting this axis may elucidate whether modulation of GDF-15 signaling provides therapeutic benefits or enhances diagnostic precision.

The development of multimarker panels including GDF-15, along with natriuretic peptides and inflammatory or metabolic biomarkers, may improve early detection of high-risk phenotypes. Also, more diverse and representative populations covering multiple races, age groups, and cardiometabolic phenotypes, are essential for improving external validity and understanding population-specific patterns.

## 5. Conclusions

Obesity, heart failure, and subclinical atherosclerosis are linked through pathogenic pathways primarily driven by inflammation, oxidative stress, endothelial damage, and metabolic disturbances. GDF-15 integrates signals from all these pathways, indicating its potential role in cardiometabolic disorder assessment. Clinical evidence establishes a correlation between GDF-15 and subclinical atherosclerosis in specific clinical context; however, no research links GDF-15 to HF, obesity and subclinical atherosclerosis.

Despite the heterogeneity and limitations of research due to the studies’ designs, cohorts, and inadequate cardiometabolic evaluation, the overall findings endorse GDF-15 as a possible biomarker for global cardiometabolic stress. Its capacity to detect metabolic, vascular, and cardiac anomalies indicates a possible use in detecting patients at elevated risk for concurrent obesity-related CVD.

Additional prospective studies are required to validate the clinical use of GDF-15 in quantifying the relationship between obesity, heart failure, and subclinical atherosclerosis, as well as to assess its significance in individualized risk estimation and disease prevention.

## Figures and Tables

**Table 1:** Joanna Briggs Institute risk of bias assessment for cross-sectional studies.

Study	Q1	Q2	Q3	Q4	Q5	Q6	Q7	Q8
Carvalho, 2018 [15]	Y	Y	Y	Y	Y	U	Y	Y
Garcia, 2024 [16]	Y	Y	Y	Y	Y	Y	Y	Y
Girona, 2025 [17]	Y	Y	Y	Y	Y	Y	Y	Y
Gopal, 2014 [18]	Y	Y	Y	Y	Y	Y	Y	Y
Guardiola, 2024 [19]	Y	Y	Y	Y	Y	Y	Y	Y
He, 2020 [20]	Y	Y	Y	Y	Y	Y	Y	Y
Kaiser, 2021 [21]	Y	Y	Y	Y	Y	Y	Y	Y
Kiss, 2023 [22]	Y	Y	Y	Y	Y	Y	Y	Y
Lind, 2009 [23]	Y	Y	Y	Y	Y	Y	Y	Y
Martinez, 2017 [24]	Y	Y	Y	Y	Y	Y	Y	Y
Royston, 2022 [25]	Y	Y	Y	Y	Y	Y	Y	Y
Tanrikulu, 2017 [26]	Y	Y	Y	Y	U	N	Y	Y
Tektonidou, 2022 [27]	Y	Y	Y	Y	Y	Y	Y	Y
Ueland, 2025 [28]	Y	Y	Y	Y	Y	Y	Y	Y

Abbreviations: Q1: Were the criteria for inclusion in the sample clearly defined?; Q2: Were the study subjects and the setting described in detail?; Q3: Was the exposure measured in a valid and reliable way?; Q4: Were objective, standard criteria used for measurement of the condition?; Q5: Were confounding factors identified?; Q6: Were strategies to deal with confounding factors stated?; Q7: Were the outcomes measured in a valid and reliable way?; Q8: Was appropriate statistical analysis used?; Y, yes; N, no; U, unclear.

**Table 2:** Joanna Briggs Institute risk of bias assessment for cohort studies.

Study	Q1	Q2	Q3	Q4	Q5	Q6	Q7	Q8	Q9	Q10	Q11
Chuang, 2025 [29]	Y	Y	Y	Y	U	U	Y	U	U	U	Y
Rohatgi, 2012 [30]	Y	Y	Y	Y	Y	U	Y	Y	Y	Y	Y
Yilmaz, 2015 [31]	Y	Y	Y	Y	Y	Y	Y	Y	U	U	Y

Abbreviations: Q1: Were the two groups similar and recruited from the same population?; Q2: Were the exposures measured similarly to assign people to both exposed and unexposed groups?; Q3: Was the exposure measured in a valid and reliable way?; Q4: Were confounding factors identified?; Q5: Were strategies to deal with confounding factors stated?; Q6: Were the groups/participants free of the outcome at the start of the study (or at the moment of exposure)?; Q7: Were the outcomes measured in a valid and reliable way?; Q8: Was the follow up time reported and sufficient to be long enough for outcomes to occur?; Q9: Was follow up complete, and if not, were the reasons to loss to follow up described and explored?; Q10: Were strategies to address incomplete follow up utilized?; Q11; Was appropriate statistical analysis used?; Y, yes; U, unclear.

**Table 3:** Joanna Briggs Institute risk of bias assessment for case–control study.

Study	Q1	Q2	Q3	Q4	Q5	Q6	Q7	Q8	Q9	Q10
Efat, 2022 [32]	Y	Y	Y	Y	Y	Y	U	Y	Y	Y

Abbreviations: Q1: Were the groups comparable other than the presence of disease in cases or the absence of disease in controls?; Q2: Were cases and controls matched appropriately?; Q3: Were the same criteria used for identification of cases and controls?; Q4: Was exposure measured in a standard, valid and reliable way?; Q5: Was exposure measured in the same way for cases and controls?; Q6: Were confounding factors identified? Q7: Were strategies to deal with confounding factors stated? Q8: Were outcomes assessed in a standard, valid and reliable way for cases and controls?; Q9: Was the exposure period of interest long enough to be meaningful?; Q10: Was appropriate statistical analysis used?; Y, yes; U, unclear.

**Table 4:** Summary of clinical studies regarding GDF-15 association with subclinical atherosclerosis in various population.

Study	Population	Atherosclerotic Marker	Results				Conclusion	Key Notes
			GDF-15– Atherosclerosis	GDF-15– Obesity/ Metabolic Profile	GDF-15– HF/CV Risk	Other Outcomes		
Metabolic disorders								
Chuang, 2025 [29]	174 patients T2DM Taiwan 20–80 y.o. 63.79% males	ABI CAVI	No association Increased baseline GDF-15 → higher risk of PAD.	No association.	Positive association.	Positive association between HF and ABI. SGLT2 inhibitors → lower levels of GDF-15.	GDF-15 serum levels are associated with PAD in patients with T2DM.	Metabolic and vascular stress
Girona, 2025 [17]	156 patients MASLD Spain 39–68 y.o. 46.8% males	CIMT	Positive association. Increased baseline GDF-15 → higher risk of atherosclerotic disease	Positive association with lipid profile.	N/A	Positive association GDF-15 with liver steatosis.	GDF-15 was correlated with atherosclerotic disease and metabolic disturbances.	
He, 2020 [20]	376 patients T2DM China 38–58 y.o. 68.8% males	FIMT	Positive association.	Positive association with BMI. Positive association with HOMA-IR. Positive association with LDL-cholesterol.	N/A	Positive association between GDF-15 and LEAD, in patients with BMI >25 kg/m^2^.	GDF-15 was associated with LEAD independent of BMI.	
Chronic inflammatory diseases								
Martinez, 2017 [24]	694 patients COPD U.S.A. 52.9% males	CACS	Positive association.	No association with BMI. Positive association with T2DM. Positive association with lipid profile.	Positive association with CV risk score.	Positive association with smoking duration.	GDF-15 can be an independent risk factor for subclinical atherosclerosis in patients with COPD without CVD.	Endothelial dysfunction is the main mechanism.
Kaiser, 2021 [21]	85 patients Psoriasis Germany ≥30 y.o. 71.8% males	CIMT CACS	Positive association.	N/A	Positive association with CVD. Positive association with CV risk score.	N/A	GDF-15 was associated with atherosclerosis disease.	
Tanrikulu, 2017 [26]	82 patients RA Turkey 18–65 y.o. 70.7% males	CIMT	Positive association.	N/A	N/A	Positive association with inflammatory markers. Positive association with disease activity.	GDF-15 could be a marker of atherosclerosis.	
Tektonidou, 2022 [27]	120 patients APS Greece 30.9% males	CIMT	Positive association.	No association.	N/A	N/A	GDF-15 could be a marker of atherosclerosis in patients with APS.	
HIV cohorts								
Carvalho, 2018 [15]	67 patients HIV Brazil 26–41 y.o. 82.1% males	CIMT	No association.	N/A	N/A	Increased prevalence of dyslipidemia. Positive association between BMI and CV risk. Age was risk factor for increased CIMT.	There was no correlation between GDF-15 and CIMT.	Systemic inflammation driven by HIV infection itself or antiretroviral treatment, enhance atherosclerosis.
Ueland, 2025 [28]	393 patients. HIV Africa 30–50 y.o. 54.2% males	CIMT	No association.	N/A	N/A	Positive association with antiretroviral treatment.	No correlation between GDF-15 and atherosclerosis.	
Royson, 2022 [25]	147 patients HIV Canada ≥40 y.o. 86% males	Coronary plaque Total plaque volume (TPV) low attenuation plaque volume (LAPV)	Positive association with TPV. Positive association with LAPV.	N/A	N/A	Increased levels in patients with HIV, independent of the presence of coronary plaque (*p* < 0.001). Positive correlation between TPV, LAPV and hsCRP in patients with HIV.	GDF-15 levels are independently associated with coronary plaque, both in HIV group and controls.	
General population/elderly								
Gopal, 2015 [18]	3111 patients U.S.A. 46% males	CIMT	Positive association with carotid plaque.	N/A	N/A	Adding GDF-15 to CRP, increase the risk of carotid plaque, but GDF-15 alone is a marker of inflammation.	In patients without CVD, GDF-15 was associated with subclinical atherosclerosis.	Chronic inflammation, aging, genetic variants and CV risk factors stimulate atherosclerosis.
Guardiola, 2024 [19]	153 patients Spain 52.2% males	CIMT	Positive association between GDF-15 variant (*rs1054564)* and atherosclerotic plaque (*p = 0.015*).	N/A	N/A	Variant carriers have an increased risk of diabetes (OR = 2.75, *p* = 0.005).	GDF-15 genetic phenotype could improve the risk stratification of metabolic disturbances and subclinical atherosclerosis development.	
Lind, 2009 [23]	1004 patients Sweeden 70 y.o. 50% males	CIMT	Positive association with carotid plaque.	Positive association.	Positive association with NT-proBNP.	Positive association with CRP.	GDF-15 is an independent biomarker useful in vascular dysfunction assessment.	
Garcia, 2024 [16]	2024 patients Germany 51.3% males	CIMT ABI	No association with ABI. No association with CIMT. Positive association with carotid plaque.	No association.	Positive association with NT-proBNP.	N/A	GDF-15 is correlated with carotid plaque, but not with CIMT.	
Kiss, 2023 [22]	269 patients Hungary 35–85 y.o. 46.5% males	CACS ABI	Positive association.	N/A	N/A	Positive association in elderly.	GDF-15 was associated with subclinical atherosclerosis in elderly patients.	
Rohatgi, 2012 [30]	2564 patients U.S.A. 30–65 y.o.	CACS	Positive association.	No association with BMI. Positive association with hsCRP. Positive association with lipid profile.	Positive association with NT-proBNP.	GDF-15 ≥1800 pg/L is a predictor for all-cause mortality and CV death.	GDF-15 was associated with subclinical atherosclerosis and mortality in a multiethnic population.	
Other diseases								
Efat, 2021 [32]	90 patients Beta-thalassemia Egypt ≥18 y.o. 42.2% males	CIMT	Positive association. GDF-15 and the GDF-15 to CIMT ratio → predictors for subclinical atherosclerosis.	No association with BMI. Positive association with lipid profile. Positive association with inflammatory markers.	N/A	Positive association with history of blood transfusion. GDF-15 ≥1440.01 pg/dL is a predictor for atherosclerosis.	GDF-15 is correlated with CIMT in patients with beta-thalassemia and blood transfusion dependence.	Vascular dysfunction enhances atherosclerosis.
Yilmaz, 2014 [31]	132 patients CKD-HD Turkey 29–84 y.o. 50.75% males	CIMT	Positive association.	No association. Positive association with CRP. Negative association with LDL-cholesterol.	N/A	Positive association with HD. Strong predictor of mortality in HD patients.	GDF-15 was associated with atherosclerosis, malnutrition and inflammation in patients with CKD and HD.	Metabolic, vascular and uremic stress from CKD stimulate atherosclerosis.

Abbreviations: ABI, ankle-brachial index; APS, antiphospholipid syndrome; BMI, body mass index; CACS, coronary artery calcium score; CAVI, cardio-ankle vascular index; CIMT, carotid intima-media thickness; CKD, chronic kidney disease; COPD, chronic obstructive pulmonary disorder; CVD, cardiovascular diseases; CV, cardiovascular; FIMT, femoral intima-media thickness; HD, hemodialysis; HIV, human immunodeficiency virus; hsCRP, high-sensitive C reactive protein; LAPV, low attenuation plaque volume; LEAD, lower extremity atherosclerotic disease; MASLD, metabolic-dysfunction associated steatotic liver disease; N/A, not available; NT-proBNP, N-terminal pro-B-type natriuretic peptide; PAD, peripheral artery disease; RA, rheumatoid arthritis; SGLT2, sodium glucose co-transporter 2; TPV, total plaque volume; T2DM, type 2 diabetes mellitus; y.o., years old.

**Table 5:** Major confounders and their impact on the association between GDF-15 and subclinical atherosclerosis, cardiovascular disorders and metabolic profile.

Study	Statistical Associations	Confounders	Observation
Metabolic disorders			
Chuang, 2025 [29]	⊗ with ABI (*p* > 0.05) ⊕ with HF (*p* = 0.022)	Age, gender, BMI, HTN, hyperlipidemia, HF, stroke, renal disease	Statistical analysis was conducted based on GDF-15’s 50th percentile and not absolute value.
Girona, 2025 [17]	⊕ with CIMT (ρ = 0.321, *p* < 0.001) ⊕ with lipid profile (VLDL-cholesterol: β = 0.475; LDL-cholesterol: β = 0.217, VLDL-triglyceride: β = 0.478; LDL-triglyceride: β = 0.503; HDL-triglyceride: β = 0.327; *p* < 0.0001) ⊖ with HDL-cholesterol (β = −0.273, *p* = 0.007)	Age, insulin therapy, oral antidiabetic therapy, oral hypotensive therapy	In crude analysis, GDF-15 showed a strong positive association with VLDL-cholesterol, VLDL-triglyceride, LDL-triglyceride, a moderate positive association with LDL-cholesterol, HDL-cholesterol, and HDL-triglycerides. After adjustment for confounders, GDF-15 remained independently associated with lipid profile remained positive for VLDL-cholesterol, VLDL-triglyceride, LDL-triglyceride (*p* < 0.001).
He, 2020 [20]	⊕ with FIMT (ρ = 0.164, *p* = 0.001) ⊕ with LEAD (OR = 1.389, CI95%: 1.136–2.242, *p* = 0.007) ⊕ with BMI (ρ = 0.179, *p* < 0.001) ⊕ with HOMA-IR (ρ = 0.103, *p* = 0.046) ⊖ with lipid profile (triglyceride: ρ = −0.214, *p* < 0.001; LDL-cholesterol: ρ = −0.206, *p* < 0.001, HDL-cholesterol: ρ = −0.142, *p* = 0.006).	BMI, gender, blood pressure, HbA1c, HOMA-IR, lipid profile, CRP, eGFR	GDF-15 showed a very weak correlation with FIMT and BMI, that remained independently positive after adjustment for confounders (FIMT: β = 0.162, *p* = 0.002 and BMI: β = 0.193, *p* < 0.001). GDF-15 showed a moderate association with LEAD in general population, that remained statistically significant after adjustment (OR = 1.419, CI95%: 1.118–1.802, *p* < 0.05), with a higher risk in patients with BMI >25 kg/m^2^ (OR = 1.582, CI95%: 1.069–2.341, *p* < 0.05).
Chronic inflammatory diseases			
Martinez, 2017 [24]	⊕ with CACS (ρ = 0.269, *p* < 0.0001) ⊗ with BMI (*p* = 0.32) ⊕ with T2DM (*p* < 0.001) ⊕ with lipid profile (*p* = 0.005)	Cardiovascular risk, comorbidities, lung function, biomarkers (NT-proBNP, troponin T, interleukin-6)	Statistical analysis was conducted based on GDF-15 tertiles. GDF-15 showed a weak positive correlation with CACS, that remained positive after adjustment.
Kaiser, 2021 [21]	⊕ with CIMT (ρ = 0.53, *p* = <0.001) ⊕ with CACS (ρ = 0.40, *p* = 0.018) ⊕ with carotid plaque (OR = 1.30, CI95%: 1.09–1.61, *p* = 0.007)	AHA risk score, hs-CRP	Although GDF-15 showed a moderate positive correlation with CIMT, the association was lost after adjustment for confounders. There was a moderate positive correlation between GDF-15 and CACS, that remained independently significant after adjustment, with higher GDF-15 levels associated with coronary atherosclerosis (OR = 1.70, CI95%: 1.34–2.33, *p* < 0.001).
Tanrikulu, 2017 [26]	⊕ with CIMT (ρ = 0.543, *p* = 0.001)	-	-
Tektonidou, 2022 [27]	⊕ with CIMT (β = 0.068, *p* = 0.006) ⊗ with BMI (*p* = 0.685)	Gender, age, renal function, treatment, adjusted global APS score for cardiovascular diseases	Statistical analysis was conducted based 1200 pg/mL cut-off level. There was a very weak association between GDF-15 and CIMT, that remained independent associated after adjustment for gender, adjusted global APS score for cardiovascular diseases (β = 0.059, CI95%: 0.008–0.110, *p* = 0.024), and treatment with hydroxychloroquine (β = 0.064, CI95%: 0.015–0.113, *p* = 0.011) and statins (β = 0.059, CI95%: 0.008–0.110, *p* = 0.025). After adjustment for age and renal function, the association is no longer statistically significant.
HIV cohorts			
Carvalho, 2018 [15]	⊗ with CIMT	-	-
Ueland, 2025 [28]	⊗ with CIMT	-	-
Royston, 2022 [25]	⊕ with total plaque volume in patients with HIV (ρ = 0.29, *p* = 0.006) and without HIV (ρ = 0.62, *p* < 0.001) ⊕ with low attenuation plaque volume in patients with HIV (ρ = 0.30, *p* = 0.005) and without HIV (ρ = 0.60, *p* < 0.001)	Age, gender, smoking, HTN, T2DM, BMI, treatment (statins)	After adjustment for confounders, in control group, GDF-15 remain positively associated with coronary atherosclerosis (OR = 35.38, CI95%: 1.19–999, *p* = 0.04). After adjustment for confounders, in cohort with HIV, GDF-15 no longer correlated with coronary atherosclerosis (OR = 1.37, CI95%: 0.65–2.90, *p* = 0.67).
General population			
Gopal, 2015 [18]	⊕ with internal carotid artery IMT (β = 0.040, *p* < 0.0001) ⊕ with carotid plaque (OR = 1.33, CI95%: 1.20–1.48, *p* < 0.0001)	Age, gender, blood pressure, HTN treatment, total and HDL cholesterol, T2DM, smoking, BMI	There was a crude small association between GDF-15 and carotid plaque that remained independently significant after adjustment for age and gender (OR = 1.48, CI95%: 1.34–1.63, *p* < 0.0001), and after multivariable analysis (β = 0.04, *p* < 0.0001), with higher GDF15 levels associated with increased odds of carotid plaque. There was a moderate correlation between GDF-15 and internal carotid artery IMT in crude analysis, that remained independently significant after adjustment for age and gender (β = 0.070, *p* < 0.0001) and multivariable analysis (OR = 1.33, CI95%: 1.20–1.48, *p* < 0.0001), with higher GDF15 levels associated with increased odds of carotid atherosclerosis.
Guardiola, 2024 [19]	⊕ with atherosclerotic plaque (OR = 2.44, CI95%: 1.19–5.03, *p* = 0.015)	Age, gender	There was a strong positive independent association in crude analysis between GDF-15 wild carrier variant and atherosclerotic plaque, that remained statistically significant after adjusting for age (OR = 2.44, CI95%: 1.11–5.37, *p* = 0.026) and gender (OR = 2.41, CI95%: 1.08–5.37, *p* = 0.032).
Lind, 2009 [23]	⊕ with CIMT (ρ = 0.11, *p* < 0.001) ⊕ with carotid plaque (ρ = 0.13, *p* < 0.001)	BMI, gender, smoking, HTN, waist circumference, T2DM, glucose, lipid profile, CRP, NT-proBNP, eGFR	Although GDF-15 showed a very weak positive correlation with CIMT in crude analysis, the association was lost after adjusting for confounders (*p* = 0.14). GDF-15 showed a very weak positive correlation with carotid plaque, that remained statistically significant after adjustment for confounders (*p* = 0.031).
Garcia, 2024 [16]	⊗ with ABI ⊗ with CIMT ⊕ with carotid plaque	Sex, age, BMI, LDL-C, diabetes, smoking status, eGFR and hypertension	GDF-15 showed a positive correlation with carotid plaque, that remained statistically positive after adjustment for confounders (β = 0.39, *p* < 0.001).
Kiss, 2023 [22]	⊕ with CACS (ρ = 0.339, *p* < 0.001) ⊕ with ABI (OR = 1.001, *p* = 0.027)	No confounders specificized.	GDF-15 showed a positive weak correlation with CACS in crude analysis, that remained statistically significant after adjustment for confounders in elderly group (β = 0.148, *p* = 0.003). GDF-15 showed a small association with ABI in crude analysis, that remained independently significant after adjustment for confounders in elderly group (β = 0.088, *p* = 0.041).
Rohatgi, 2012 [30]	⊕ with CACS (*p < 0.0001*) ⊗ with BMI (β = −0.02, *p* = 0.27) ⊖ with lipid profile (LDL-cholesterol: β = −0.10, *p < 0.0001*; cholesterol: β = −0.08, *p* < 0.0001)	Age, gender, black race, HTN, T2DM, smoking, left ventricular mass, lipid profile	There was a positive association between GDF-15 and CACS in crude analysis, that remained statistically significant after adjustment for CRP, NT-proBNP, and cardiac troponin T, GDF-15 ≥1800 ng/L being associated with CAC >10 (OR = 2.1, CI95%: 1.2–3.7, *p* = 0.01), CAC ≥100 (OR = 2.6, CI95%: 1.4–4.9, *p* = 0.002).
Other diseases			
Efat, 2021 [32]	⊕ with CIMT (*p* < 0.001) ⊗ with BMI (ρ = 0.073, *p* = 577) ⊕ with cholesterol (ρ = 0.365, *p* = 0.004)	Smoking, ferritin, blood transfusion, lipid profile.	There was a positive association between GDF-15 and CIMT in crude analysis, that remained independently significant after adjustment, GDF-14 ≥1839.89 pg/mL being associated with increased dds of carotid atherosclerosis (OR = 62.143, CI95%: 5.780–66.166, *p* = 0.001).
Yilmaz, 2014 [31]	⊕ with CIMT (ρ = 0.607, *p* < 0.001) ⊗ with BMI (ρ = −0.014, *p* = 0.958) ⊕ with CRP (ρ = 0.250, *p* < 0.010) ⊖ with LDL-cholesterol (ρ = −0.237, *p* = 0.020).	Age, CRP, T2DM, gender, albumin, BMI	GDF-15 showed a strong correlation with CIMT in crude analysis, that remained independently significant after adjustment for confounders, GDF-15 being a strong independent predictor for mortality (HR = 5.65, *p* < 0.01).

Abbreviations: ⊕, positive association; ⊖, negative association; ⊗, no association; ABI, ankle-brachial index; APS, antiphospholipid syndrome; BMI, body mass index; CACS, coronary artery calcium score; CI, confidence interval; CIMT, carotid intima-media thickness; eGFR, estimated glomerular filtration rate; FIMT, femoral intima-media thickness; GDF-15, growth differentiation factor; HbA1c, hemoglobin A1c; HDL, high density lipoprotein; HF, heart failure, HIV, human immunodeficiency virus; HOMA-IR, Homeostasis Model Assessment of Insulin Resistance; hs-CRP, high-sensitive C reactive protein; HTN, arterial hypertension; IMT, intima media thickness; LDL-cholesterol, low density lipoprotein cholesterol; NT-proBNP, N-terminal pro-B-type natriuretic peptide; OR, odds ratio; T2DM, type 2 diabetes mellitus; VLDL-cholesterol, very low density lipoprotein cholesterol.

**Table 6:** Studies’ limitations.

Study	Limitations
Metabolic disorders	
Chuang ^1^, 2025 [29]	The result cannot be generalized to others ethnic groups. SGLT2 inhibitors may interfere with GDF-15. No confounding variables were assessed.
Girona ^1^, 2025 [17]	MASLD was assessed using non-invasive techniques. No information about HF.
He ^1^, 2020 [20]	No evaluation of ABI or symptoms. Body composition differs in Asia compared to other regions, so the results cannot be generalized into general population. No information about BMI, just associations with metabolic profile. No information about HF.
Chronic inflammatory diseases	
Martinez ^1^, 2017 [24]	The study did not compare GDF-15 and CACS between obese vs. normal-weight patients. Not all diseases were proved by medical records (patients self-reported history).
Kaiser ^1^, 2021 [21]	No healthy control group included. The patients included had different disease activity and different treatments. No other vascular assessment included.
Tanrikulu ^1^, 2017 [26]	No information about HF. No correlation between GDF-15 and obesity.
Tektonidou ^1^, 2022 [27]	APS is not a frequent disease into general population.
HIV cohorts	
Carvalho ^1^, 2018 [15]	Young population, without CVD. Increased prevalence of dyslipidemia could be explained by antiviral therapy.
Ueland ^1^, 2025 [33]	Common CIMT evaluation only. No viremia was assessed during the study. Antiviral therapy duration was not assessed.
Royston ^1^, 2022 [25]	Most of the patients had metabolic syndrome or its components. There was not specified the HIV treatment and its implication into results.
General population	
Gopal ^1^, 2015 [18]	The results could be generalized to white populations only. Incomplete atherosclerotic disease evaluation. No information about HF. No information about BMI.
Guardiola ^1^, 2024 [19]	No available data regarding glycemic control status. No information about BMI. No information about HF.
Lind ^1^, 2009 [23]	The results cannot be generalized. Not all CVD were proved by medical records (patients self-reported history such as HF or angina).
Garcia ^1^, 2024 [16]	Large number of proteins assessed with cardiovascular features. No data regarding HF patients, just with NT-proBNP values.
Kiss ^1^, 2023 [22]	The study sample was formed by Caucasian patients, so the result cannot be generalized. No information about HF. No information about metabolic profile and GDF-15.
Rohatgi ^2^, 2012 [30]	Limited statistical power due to young population. Not all diseases were proved by medical records (patients self-reported history).
Other diseases	
Efat ^3^, 2021 [32]	Young population, with blood transfusion dependence.
Yilmaz ^2^, 2014 [31]	No assessment of malnutrition, which can interfere with GDF-15. No information about HF.

Abbreviations: ABI, ankle-brachial index; BMI, body mass index; CACS, coronary artery calcium score; CIMT, carotid intima-media thickness; CVD, cardiovascular diseases; GDF-15, growth differentiation factor 15; HIV, human immunodeficiency virus; HF, heart failure; MASLD, metabolic associated steatotic liver disease; NT-proBNP, N-terminal pro-B-type natriuretic peptide; SGLT2, sodium glucose co-transporter 2; y.o., years old. ^1^ cross-sectional study; ^2^ cohort study; ^3^ case–control study.

## Data Availability

No new data were created or analyzed in this study. Data sharing is not applicable to this article.

## References

[1] Asrih M., Wei S., Nguyen T.T., Yi H., Ryu D., Gariani K. (2023). Overview of Growth Differentiation Factor 15 in Metabolic Syndrome. J. Cell. Mol. Med..

[2] Manta E., Iliakis P., Fragoulis C., Leontsinis I., Stamoulopoulos I., Chrysohoou C., Tsioufis K. (2025). Tracking Pathways Linking Obesity with Heart Failure. Nutrients.

[3] Dronkers J., Van Veldhuisen D.J., Van Der Meer P., Meems L.M.G. (2024). Heart Failure and Obesity. J. Am. Coll. Cardiol..

[4] Koskinas K.C., Van Craenenbroeck E.M., Antoniades C., Blüher M., Gorter T.M., Hanssen H., Marx N., McDonagh T.A., Mingrone G., Rosengren A. (2024). Obesity and Cardiovascular Disease: An ESC Clinical Consensus Statement. Eur. Heart J..

[5] Ng M., Fleming T., Robinson M., Thomson B., Graetz N., Margono C., Mullany E.C., Biryukov S., Abbafati C., Abera S.F. (2014). Global, Regional, and National Prevalence of Overweight and Obesity in Children and Adults during 1980–2013: A Systematic Analysis for the Global Burden of Disease Study 2013. Lancet.

[6] Vila G., Riedl M., Anderwald C., Resl M., Handisurya A., Clodi M., Prager G., Ludvik B., Krebs M., Luger A. (2011). The Relationship between Insulin Resistance and the Cardiovascular Biomarker Growth Differentiation Factor-15 in Obese Patients. Clin. Chem..

[7] Desmedt S., Desmedt V., De Vos L., Delanghe J.R., Speeckaert R., Speeckaert M.M. (2019). Growth Differentiation Factor 15: A Novel Biomarker with High Clinical Potential. Crit. Rev. Clin. Lab. Sci..

[8] Tian T., Liu M., Little P.J., Strijdom H., Weng J., Xu S. (2025). Emerging Roles of GDF15 in Metabolic and Cardiovascular Diseases. Research.

[9] Li J., Hu X., Xie Z., Li J., Huang C., Huang Y. (2024). Overview of Growth Differentiation Factor 15 (GDF15) in Metabolic Diseases. Biomed. Pharmacother..

[10] di Candia A.M., de Avila D.X., Moreira G.R., Villacorta H., Maisel A.S. (2021). Growth Differentiation Factor-15, a Novel Systemic Biomarker of Oxidative Stress, Inflammation, and Cellular Aging: Potential Role in Cardiovascular Diseases. Am. Heart J. Plus.

[11] Pence B.D. (2022). Growth Differentiation Factor-15 in Immunity and Aging. Front Aging.

[12] Guo J., Zhang Z. (2025). Novel Biomarker Panel Combined with Imaging Parameters for Predicting Cardiovascular Complications in Diabetic Patients: A Retrospective Cohort Study. BMC Cardiovasc. Disord..

[13] Page M.J., McKenzie J.E., Bossuyt P.M., Boutron I., Hoffmann T.C., Mulrow C.D., Shamseer L., Tetzlaff J.M., Akl E.A., Brennan S.E. (2021). The PRISMA 2020 Statement: An Updated Guideline for Reporting Systematic Reviews. BMJ.

[14] Aromataris E., Lockwood C., Porritt K., Pilla B., Jordan Z., JBI (2024). Manual for Evidence Synthesis.

[15] Carvalho P.V.D.C., Caporali J.F.D.M., Vieira É.L.M., Guimarães N.S., Fonseca M.O., Tupinambás U. (2018). Evaluation of Inflammatory Biomarkers, Carotid Intima-Media Thickness and Cardiovascular Risk in HIV-1 Treatment-Naive Patients. Rev. Soc. Bras. Med. Trop..

[16] Garcia T., Petrera A., Hauck S.M., Baber R., Wirkner K., Kirsten H., Pott J., Tönjes A., Henger S., Loeffler M. (2024). Relationship of Proteins and Subclinical Cardiovascular Traits in the Population-Based LIFE-Adult Study. Atherosclerosis.

[17] Girona J., Guardiola M., Barroso E., García-Altares M., Ibarretxe D., Plana N., Ribalta J., Amigó N., Correig X., Vázquez-Carrera M. (2025). GDF15 Circulating Levels Are Associated with Metabolic-Associated Liver Injury and Atherosclerotic Cardiovascular Disease. Int. J. Mol. Sci..

[18] Gopal D.M., Larson M.G., Januzzi J.L., Cheng S., Ghorbani A., Wollert K.C., Kempf T., D’Agostino R.B., Polak J.F., Ramachandran V.S. (2014). Biomarkers of Cardiovascular Stress and Subclinical Atherosclerosis in the Community. Clin. Chem..

[19] Guardiola M., Girona J., Barroso E., García-Altares M., Ibarretxe D., Plana N., Ribalta J., Correig X., Vázquez-Carrera M., Masana L. (2024). The GDF15 3′ UTR Polymorphism Rs1054564 Is Associated with Diabetes and Subclinical Atherosclerosis. Int. J. Mol. Sci..

[20] He X., Su J., Ma X., Lu W., Zhu W., Wang Y., Bao Y., Zhou J. (2020). The Association between Serum Growth Differentiation Factor 15 Levels and Lower Extremity Atherosclerotic Disease Is Independent of Body Mass Index in Type 2 Diabetes. Cardiovasc. Diabetol..

[21] Kaiser H., Wang X., Kvist-Hansen A., Krakauer M., Gørtz P.M., McCauley B.D., Skov L., Becker C., Hansen P.R. (2021). Biomarkers of Subclinical Atherosclerosis in Patients with Psoriasis. Sci. Rep..

[22] Kiss L.Z., Nyárády B.B., Pállinger É., Lux Á., Jermendy Á.L., Csobay-Novák C., Soós P., Szelid Z., Láng O., Kőhidai L. (2023). Association of Growth and Differentiation Factor-15 with Coronary Artery Calcium Score and Ankle-Brachial Index in a Middle-Aged and Elderly Caucasian Population Sample Free of Manifest Cardiovascular Disease. GeroScience.

[23] Lind L., Wallentin L., Kempf T., Tapken H., Quint A., Lindahl B., Olofsson S., Venge P., Larsson A., Hulthe J. (2009). Growth-Differentiation Factor-15 Is an Independent Marker of Cardiovascular Dysfunction and Disease in the Elderly: Results from the Prospective Investigation of the Vasculature in Uppsala Seniors (PIVUS) Study. Eur. Heart J..

[24] Martinez C.H., Nelson J.D., Murray S., Wang X., Budoff M.J., Dransfield M.T., Hokanson J.E., Kazerooni E.A., Kinney G.L., Regan E.A. (2017). GDF-15 Plasma Levels in Chronic Obstructive Pulmonary Disease Are Associated with Subclinical Coronary Artery Disease. Respir. Res..

[25] Royston L., Isnard S., Perrin N., Sinyavskaya L., Berini C., Lin J., Trottier B., Baril J.-G., Chartrand-Lefebvre C., Tremblay C. (2022). Growth Differentiation Factor-15 as a Biomarker of Atherosclerotic Coronary Plaque: Value in People Living with and without HIV. Front. Cardiovasc. Med..

[26] Tanrıkulu O., Sarıyıldız M.A., Batmaz İ., Yazmalar L., Polat N., Kaplan İ., Çevik R. (2017). Serum GDF-15 Level in Rheumatoid Arthritis: Relationship with Disease Activity and Subclinical Atherosclerosis. Acta Reumatol. Port..

[27] Tektonidou M.G., Papassotiriou I., Sfikakis P.P. (2021). Growth Differentiation Factor 15 (GDF-15) as Potential Cardiovascular Risk Biomarker in Antiphospholipid Syndrome. Rheumatology.

[28] Ueland T., Nkele I., Hoel H., Lockman S., Michelsen A.E., Moshomo T., Aukrust P., Mohammed T., Trøseid M., Mosepele M. (2025). Markers of Extracellular Matrix Degradation and Inflammasome Activation Are Associated with Carotid Plaques in Virally Suppressed People with HIV in Botswana. AIDS.

[29] Chuang W.-C., Chu C.-H., Yao C.-S., Wei M.-C., Hsieh I.-L., Liao C.-M. (2025). The Value of Growth Differentiation Factor 15 as a Biomarker for Peripheral Artery Disease in Diabetes Patients. Diabetol. Metab. Syndr..

[30] Rohatgi A., Patel P., Das S.R., Ayers C.R., Khera A., Martinez-Rumayor A., Berry J.D., McGuire D.K., De Lemos J.A. (2012). Association of Growth Differentiation Factor-15 with Coronary Atherosclerosis and Mortality in a Young, Multiethnic Population: Observations from the Dallas Heart Study. Clin. Chem..

[31] Yilmaz H., Çelik H.T., Gurel O.M., Bilgic M.A., Namuslu M., Bozkurt H., Ayyildiz A., Inan O., Bavbek N., Akcay A. (2015). Increased Serum Levels of GDF-15 Associated with Mortality and Subclinical Atherosclerosis in Patients on Maintenance Hemodialysis. Herz.

[32] Efat A., Wahb R., Shoeib S.A.A., Dawod A.A.E., Abd ElHafez M.A., Abd ElMohsen E.A., Elkholy A. (2022). GDF-15 Is Associated with Atherosclerosis in Adults with Transfusion-dependent Beta-thalassemia. eJHaem.

[33] Adela R., Banerjee S.K. (2015). GDF-15 as a Target and Biomarker for Diabetes and Cardiovascular Diseases: A Translational Prospective. J. Diabetes Res..

[34] Nyárády B.B., Kiss L.Z., Bagyura Z., Merkely B., Dósa E., Láng O., Kőhidai L., Pállinger É. (2024). Growth and Differentiation Factor-15: A Link between Inflammaging and Cardiovascular Disease. Biomed. Pharmacother..

[35] Oppong R., Orru V., Marongiu M., Qian Y., Sidore C., Delitala A., Orru M., Mulas A., Piras M.G., Morrell C.H. (2025). Age-Associated Increase in Growth Differentiation Factor 15 Levels Correlates With Central Arterial Stiffness and Predicts All-Cause Mortality in a Sardinian Population Cohort. J. Am. Heart Assoc..

[36] Wang D., Day E.A., Townsend L.K., Djordjevic D., Jørgensen S.B., Steinberg G.R. (2021). GDF15: Emerging Biology and Therapeutic Applications for Obesity and Cardiometabolic Disease. Nat. Rev. Endocrinol..

[37] Ceasovschih A., Șorodoc V., Covantsev S., Balta A., Uzokov J., Kaiser S., Almaghraby A., Lionte C., Stătescu C., Sascău R. (2024). Electrocardiogram Features in Non-Cardiac Diseases: From Mechanisms to Practical Aspects. J. Multidiscip. Healthc..

[38] Fichtner K., Kalwa H., Lin M.-M., Gong Y., Müglitz A., Kluge M., Krügel U. (2024). GFRAL Is Widely Distributed in the Brain and Peripheral Tissues of Mice. Nutrients.

[39] Hsu J.-Y., Crawley S., Chen M., Ayupova D.A., Lindhout D.A., Higbee J., Kutach A., Joo W., Gao Z., Fu D. (2017). Non-Homeostatic Body Weight Regulation through a Brainstem-Restricted Receptor for GDF15. Nature.

[40] Tsai V.W.W., Husaini Y., Sainsbury A., Brown D.A., Breit S.N. (2018). The MIC-1/GDF15-GFRAL Pathway in Energy Homeostasis: Implications for Obesity, Cachexia, and Other Associated Diseases. Cell Metab..

[41] May B.M., Pimentel M., Zimerman L.I., Rohde L.E. (2021). GDF-15 as a Biomarker in Cardiovascular Disease. Arq. Bras. Cardiol..

[42] Dakota I., Wijayanto M.A., Nugrahani A.S.D., Sunjaya A.F., Rachmayanti S., Indah E.Y., Naomi N., Huang W., Tristan C.D., Fauziah M. (2025). Diagnostic and Prognostic Implications of Growth Differentiation Factor 15 in Heart Failure with Preserved Ejection Fraction: A Systematic Review and Meta-Analysis. PeerJ.

[43] Ortolá R., García-Esquinas E., Buño-Soto A., Sotos-Prieto M., Struijk E.A., Caballero F.F., Lopez-Garcia E., Banegas J.R., Rodríguez-Artalejo F. (2021). Healthy Dietary Patterns Are Associated with Lower Concentrations of Growth Differentiation Factor 15 in Older Adults. Am. J. Clin. Nutr..

[44] Banerjee D., Mani A. (2025). Obesity’s Systemic Impact: Exploring Molecular and Physiological Links to Diabetes, Cardiovascular Disease, and Heart Failure. Front. Endocrinol..

[45] Echouffo-Tcheugui J.B., Daya N., Ndumele C.E., Matsushita K., Hoogeveen R.C., Ballantyne C.M., Coresh J., Shah A.M., Selvin E. (2022). Diabetes, GDF-15 and Incident Heart Failure: The Atherosclerosis Risk in Communities Study. Diabetologia.

[46] Liuizė A., Mongirdienė A. (2024). TGF-β Isoforms and GDF-15 in the Development and Progression of Atherosclerosis. Int. J. Mol. Sci..

[47] Kittleson M.M., Benjamin E.J., Blumer V., Harrington J., Januzzi J.L., McMurray J.J.V., Vest A.R. (2025). 2025 ACC Scientific Statement on the Management of Obesity in Adults With Heart Failure. J. Am. Coll. Cardiol..

[48] Shahid I., Zakaria F., Chang R., Javed U., Amin Z.M., Al-Kindi S., Nasir K., Javed Z. (2025). Obesity and Atherosclerotic Cardiovascular Disease: A Review of Social and Biobehavioral Pathways. Methodist. Debakey Cardiovasc. J..

[49] Sakamoto D., Matsuoka Y., Nakatani D., Okada K., Sunaga A., Kida H., Sato T., Kitamura T., Tamaki S., Seo M. (2025). Role and Prognostic Value of Growth Differentiation Factor 15 in Patient of Heart Failure with Preserved Ejection Fraction: Insights from the PURSUIT-HFpEF Registry. Open Heart.

[50] Madaudo C., Coppola G., Parlati A.L.M., Corrado E. (2024). Discovering Inflammation in Atherosclerosis: Insights from Pathogenic Pathways to Clinical Practice. Int. J. Mol. Sci..

[51] Rayner J.J. (2025). Obesity and Heart Failure: Exploring the Cardiometabolic Axis. Cardiovasc. Res..

[52] Sawalha K., Norgard N.B., Drees B.M., López-Candales A. (2023). Growth Differentiation Factor 15 (GDF-15), a New Biomarker in Heart Failure Management. Curr. Heart Fail. Rep..

[53] Takaoka M., Tadross J.A., Al-Hadithi A.B.A.K., Zhao X., Villena-Gutiérrez R., Tromp J., Absar S., Au M., Harrison J., Coll A.P. (2024). GDF15 Antagonism Limits Severe Heart Failure and Prevents Cardiac Cachexia. Cardiovasc. Res..

[54] Reyes J., Yap G.S. (2023). Emerging Roles of Growth Differentiation Factor 15 in Immunoregulation and Pathogenesis. J. Immunol..

